# The Fd-GOGAT1 mutant gene *lc7* confers resistance to *Xanthomonas oryzae* pv. *Oryzae* in rice

**DOI:** 10.1038/srep26411

**Published:** 2016-05-23

**Authors:** Honglin Chen, Chunrong Li, Liping Liu, Jiying Zhao, Xuzhen Cheng, Guanghuai Jiang, Wenxue Zhai

**Affiliations:** 1 Institute of Genetics and Developmental Biology, Chinese Academy of Sciences, Beijing 100101, China; 2 National Key Facility for Crop Gene Resources and Genetic Improvement, Institute of Crop Science, Chinese Academy of Agricultural Sciences, Beijing, 100081, China; 3 State Key Laboratory of Agrobiotechnology, College of Biological Sciences, China Agricultural University, Beijing, 100193, China

## Abstract

Disease resistance is an important goal of crop improvement. The molecular mechanism of resistance requires further study. Here, we report the identification of a rice leaf color mutant, *lc7*, which is defective in chlorophyll synthesis and photosynthesis but confers resistance to *Xanthomonas oryzae* pv. *Oryzae* (*Xoo*). Map-based cloning revealed that *lc7* encodes a mutant ferredoxin-dependent glutamate synthase1 (Fd-GOGAT1). Fd-GOGAT1 has been proposed to have great potential for improving nitrogen-use efficiency, but its function in bacterial resistance has not been reported. The *lc7* mutant accumulates excessive levels of ROS (reactive oxygen species) in the leaves, causing the leaf color to become yellow after the four-leaf stage. Compared to the wild type, *lc7* mutants have a broad-spectrum high resistance to seven *Xoo* strains. Differentially expressed genes (DEGs) and qRT-PCR analysis indicate that many defense pathways that are involved in this broad-spectrum resistance are activated in the *lc7* mutant. These results suggest that Fd-GOGAT1 plays an important role in broad-spectrum bacterial blight resistance, in addition to modulating nitrogen assimilation and chloroplast development.

Bacterial blight (BB) is a serious disease in rice that is caused by the Gram-negative bacterium *Xanthomonas oryzae* pv. *oryzae* (*Xoo*) and can cause yield losses of up to 50%^1^. *Xoo* is a serious threat to agriculture and global food security^2^. The use of resistance genes in breeding programs has been regarded as the most effective and economical strategy for controlling bacterial blight. To date, a total of 38 BB resistance genes (*R* genes) have been identified^3^. Of these genes, eight BB resistance genes, namely *Xa21*^4^, *Xa1*^5^, *Xa26*^6^, *xa5*^7^,^8^, *xa13*^9^, *Xa27*^10^, *Xa10*^11^ and *Xa23*^12^, have been cloned, and the protein structures that they encode are diverse. This diversity indicates that the molecular mechanism of BB resistance is very complicated in rice. In addition, all of these genes are race specific, which restricts their direct use in rice production as the single resistance resource. A rice variety containing one of these genes can easily lose resistance owing to the rapid variation of the cognate pathological race. The incorporation of multiple resistance genes into rice cultivars is a very effective method to increase rice resistance^13^. However, gene pyramiding is a very tedious and complicated process. Plants have evolved complex resistance mechanisms to fend off microbial pathogens, in addition to these *R* genes, providing several layers of constitutive and inducible defenses. Many of these defenses are controlled by development- or metabolism-related genes that trigger a long lasting systemic acquired resistance (SAR) to defend the plant against a broad spectrum of pathogens.

Glutamate synthase (GOGAT) is a key enzyme in the synthesis of glutamate. Glutamate is a central molecule in amino acid metabolism in higher plants^3^ and is the precursor of chlorophyll synthesis in leaves^14^. There are two forms of glutamate synthase in plants that differ in electron donor: NADH-GOGAT requires pyridine nucleotides, and Fd-GOGAT requires reduced ferredoxin (Fd)^15^. NADH-GOGAT is located primarily in the plastids of non-photosynthetic tissues, such as roots, and Fd-GOGAT is located primarily in leaf chloroplasts^16^,^17^. The major role of Fd-GOGAT is the re-assimilation of the ammonium that is liberated during photorespiration^18^. In *Arabidopsis thaliana*, Fd-GOGAT is encoded by *GLU1* (Fd-GOGAT1, At5g04140) and *GLU2* (Fd-GOGAT2, At2g41220), which exhibit contrasting expression patterns. Fd-GOGAT1 is highly expressed in leaves, whereas Fd-GOGAT 2 is mostly expressed in roots. Fd-GOGAT 1 contributes the most to glutamate synthase activity^19^. In rice, there are two NADH-dependent and two ferredoxin (Fd)-dependent GOGATs^17^,^20^. Although the amino acid sequence of OsFd-GOGAT2 is not comparable to that of *Arabidopsis thaliana*, OsFd-GOGAT1 is homologous to GLU1 and GLU2 and is expressed in most tissues, especially in the leaf and leaf sheath.

Our data indicate that glutamate is a central molecule in ROS metabolism in higher plants. The mutation of OsFd-GOGAT1 increases the level of ROS and confers upon rice broad-spectrum resistance to BB.

## Results

### Characterization of the *lc7* mutant

**Figure 1:**
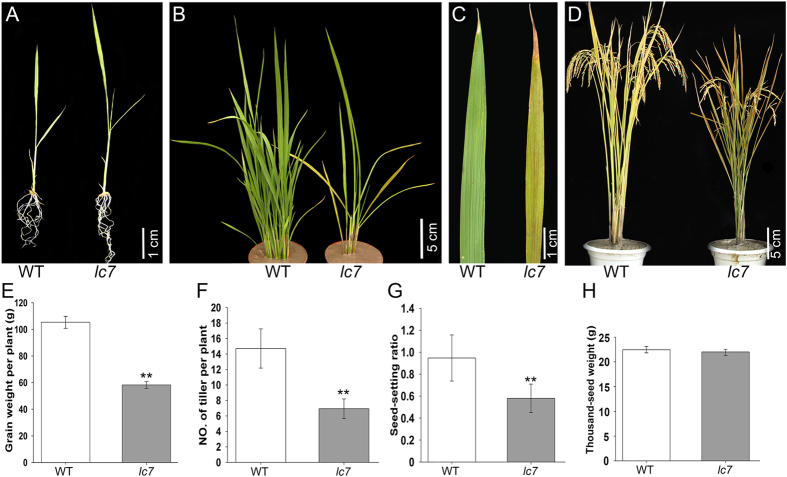
Phenotype of the *lc7* mutant. (**A**) Plant at the seedling stage. (**B**) Plant at the seedling stage grown in the field for two months. (**C**) Plant at the tillering stage. (**D**) Plant at maturity. (**E**–**G**). The investigation of agronomic traits, including grain weight per plant, tiller number seed-setting ratio, and thousand-grain weight. Asterisks indicate the significance of differences between wild-type and *lc7* mutant plants as determined by Student’s t-test. These data were obtained from three independent replicates, **P < 0.01 (*t*-test). Each scale bar is indicated.

Leaf color not only is an important identification characteristic but also plays an important role in the development and yield of rice. To study the physiological and biochemical mechanisms of leaf color, we previously generated, through Co^21^ radiation, a large rice mutation population and identified the novel chloroplast-deficient mutant *lc7.* The *lc7* mutant is characterized by yellow leaves with some brown streaks at the four-leaf stage that gradually spread over the entire leaf surface during development. At the seedling stage, the *lc7* mutants are slightly larger than those of the wild-type, and the *lc7* mutant shows a leaf color similar to that of wild-type (Fig. 1A). However, after the four-leaf stage, the wild-type leaves are larger than those of the *lc7* mutants, and the leaf color of the *lc7* mutant begins to turn yellow (Fig. 1B–D). Compared to the wild-type Nipponbare, other characteristic indexes, such as the plant height, tiller number, seed-setting ratio and thousand-grain weight, were significantly affected (Fig. 1E–H).

### *lc7* mutant is defective in chlorophyll synthesis and photosynthesis

**Figure 2:**
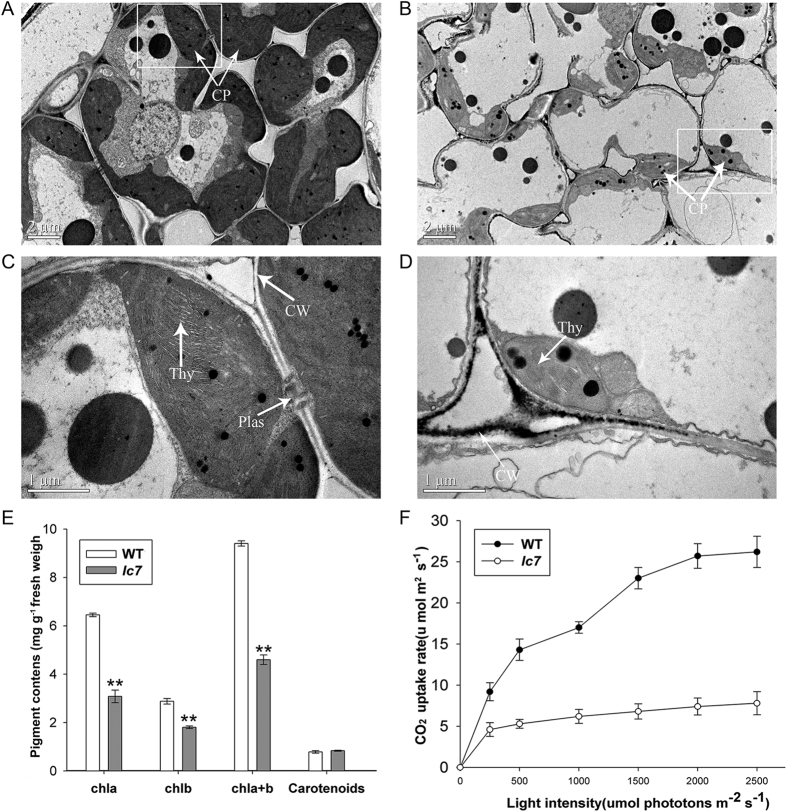
The ultrastructure and physiological-biochemical analysis of chloroplasts. (**A**,**C**) The chloroplast ultrastructure of wild-type and *lc7* at maturity. (**B**,**D**) The chloroplast structure of the *lc7* mutant at maturity. (**E**) The pigment contents of chla, chlb, and chla+chlb; the rate of chla/chlb; and the contents of carotenoids. (**F**) The photosynthetic rate of the wild-type and *lc7* at the four-leaf stage. These data were obtained from three independent replicates. *P < 0.05, **P < 0.01 (*t*-test).

The yellow color of the *lc7* mutant leaf caused us to speculate that there were some changes in its chlorophyll content or chloroplast structure. Transmission electron microscope (TEM) observation indicated that the mesophyll cell and chloroplast structures of *lc7* were abnormal. Compared to those of the wild-type (Fig. 2A,C), the volume and number of chloroplasts in *lc7* were low, lacking any lamellar structures and even chlorophyll in some cells (Fig. 2B,D). The chlorophyll a and b contents of the *lc7* mutant significantly decreased, and the total amount of chlorophyll of *lc7* was half that of the wild-type, but there was no significant difference in the carotenoid content or the ratio of chlorophyll a and b (Fig. 2E). The decreased chlorophyll content influenced the photosynthesis rate in the *lc7* mutant, consistent with its yellowish leaf phenotype (Fig. 2F), suggesting that the mutation of *lc7* impaired the development of chloroplasts.

### *lc7* activates the gene expression in the ROS scavenging pathway

**Figure 3:**
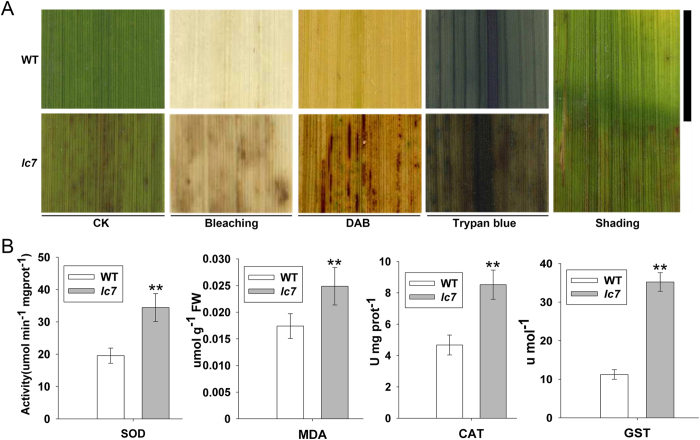
Active oxygen-scavenging enzyme activity in the *lc7* mutant. (**A**) DAB and Trypan blue staining for H_2_O_2_ accumulation and cell death. Leaves were treated at 30 days after sowing. (**B**) The assay of oxygen scavenging enzyme activity in the *lc7* mutant. SOD, superoxide dismutase; MDA, malondialdehyde; CAT, catalase; GST, glutathione-S-transferase. These data were obtained from three independent replicates. *P < 0.05, **P < 0.01 (t-test).

Reactive oxygen species (ROS) are messenger molecules and inducers of oxidative damage; they have been implicated in the regulation of innate immunity in plants. We examined the histochemical characteristics of *lc7* mutant and wild-type plants. DAB staining indicated that significant H_2_O_2_ accumulation occurred in the *lc7* mutant, and the H_2_O_2_ content was positively correlated with brown streak formation. Interestingly, the leaves of the *lc7* mutant plants did not stain with trypan blue, indicating that no membrane damage or cell necrosis occurred in *lc7* leaves. We also performed decolorizing and shading treatments on the *lc7* leaves. The brown spots of the yellow leaves were still visible after decolorizing; however, no spots were detected on the shaded leaves (Fig. 3A). In addition, the enzyme activity of the reactive oxygen scavenging system, such as SOD, MDA, CAT and GST, was typically induced in *lc7* mutants compared to the wild-type (Fig. 3B). These results suggest that an excessive accumulation of ROS but not cell death occurred in the *lc7* mutant leaves and that light was involved in the formation of yellow leaves with brown streaks. We speculated that the *lc7* gene may be involved in disease resistance.

### The *lc7* mutant shows a broad-spectrum disease resistance to bacterial blight

**Figure 4:**
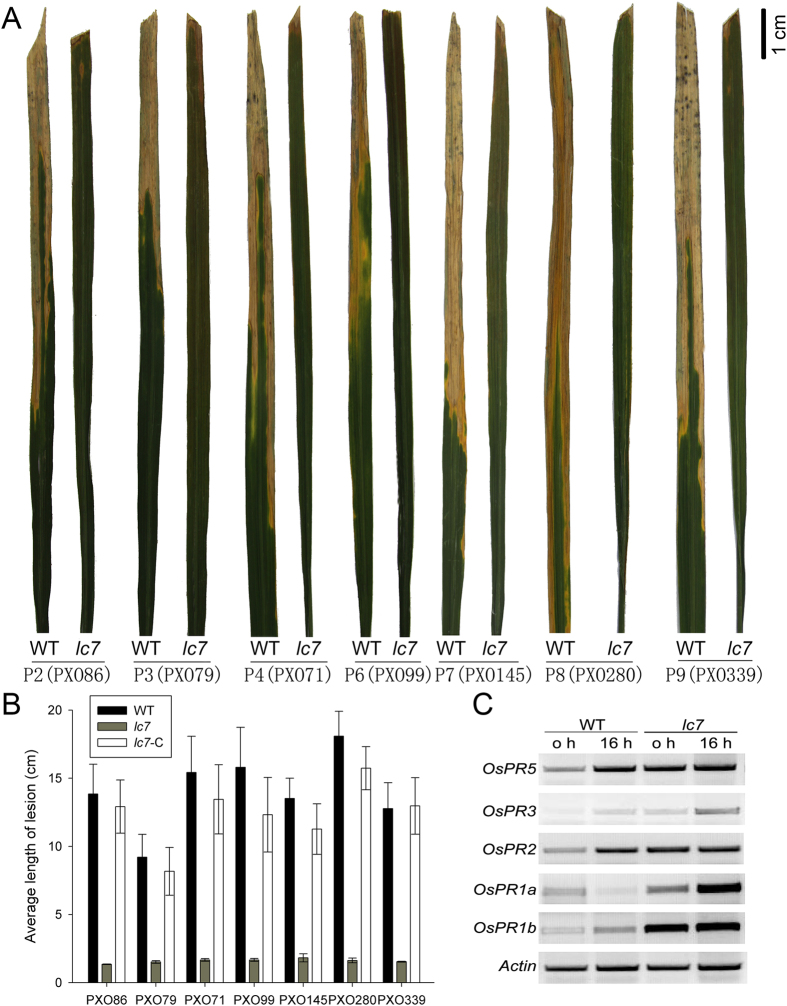
The *lc7* mutants showed high broad-spectrum resistance to *Xoo*. (**A**) Resistance phenotype of the *lc7* mutants 14 days after inoculation with seven *Xoo* strains. (**B**) Average lesion length of the *lc7* mutants 14 days after inoculation with seven *Xoo* strains. (**C**) RT-PCR analysis of pathogen-related genes (PRs) in the *lc7* mutant, *OsActin* was used as an internal control. These data were obtained from three independent replicates. The scale bar is indicated.

Because the *lc7* mutant can activate the ROS scavenging pathway and the expression of defense-related genes, we wondered whether the *lc7* mutant also confers disease resistance to pathogens. We inoculated *lc7* mutant leaves with 10 *Xanthomonas oryzae* pv. *oryzae* (*Xoo*) strains approximately two months after sowing. Compared to the wild-type, the *lc7* mutants showed a broad-spectrum high resistance to seven of these strains (PXO86, PXO79, PXO71, PXO99, PXO145, PXO280 and PXO339). Twenty days after inoculation, the average lesion length on the wild-type leaves was 13.9 cm, 9.2 cm, 15.4 cm, 15.8 cm, 13.5 cm, 18.1 cm and 12.8 cm, respectively, whereas that of the *lc7* mutants was 1.4 cm, 1.5 cm, 1.7 cm, 1.7 cm, 1.8 cm, 1.6 cm and 1.5 cm, respectively. (Table 1 and Fig. 4A,B). These data indicate that the pathogen defense response was dramatically activated in *lc7* mutant plants. In addition, we examined the expression of pathogen-related genes (PR genes), and found that five of these genes, including *OsPR1a*, *OsPR1b*, *OsPR2*, *OsPR3*, and *OsPR5*, were greatly induced in the *lc7* mutant after challenge with *Xoo* strain PXO99 when the leaf became yellow and when the brown-streak phenotype appeared (Fig. 4C), indicating that disease resistance was activated in the *lc7* mutant.

### Map-based cloning of *lc7*

**Figure 5:**
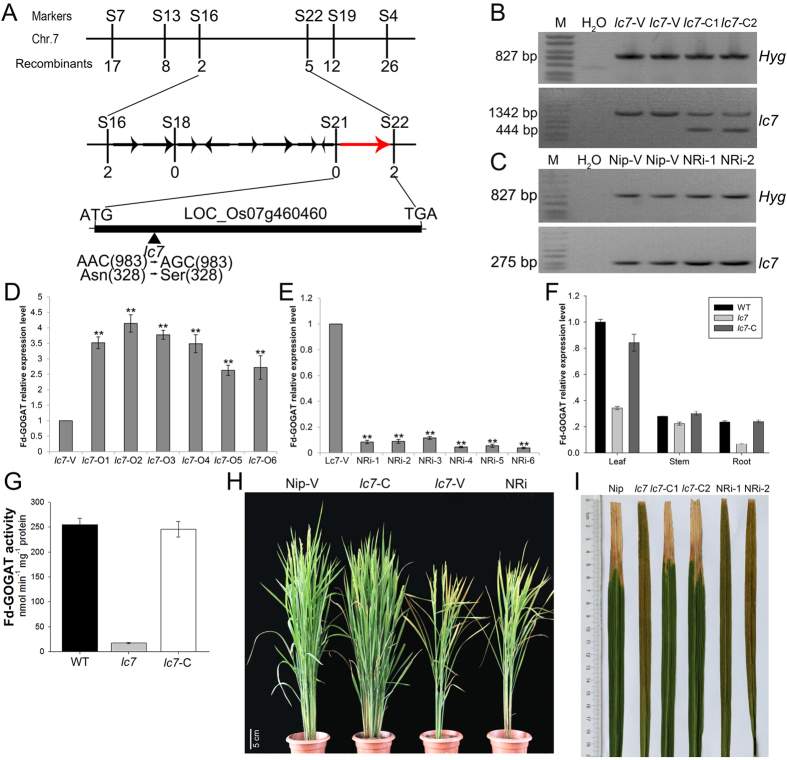
Map-based cloning of the *lc7* gene. (**A**) The *lc7* gene was mapped to a 78-kb region between the markers S16 and S22 on the long arm of chromosome 7 with nine candidate genes. The *LOC_Os07g46460* gene encoding Fd-GOGAT1 in the *lc7* mutant with a single-base substitution at the 983^rd^ position is the best candidate gene of *lc7*. (**B**,**C**) PCR amplification of Hyg and LC7 from the control and transgenic plants. M = Trans2k plus DNA Marker (http://www.transgen.com.cn). (**D**,**E**) The relative expression level of *Fd-GOGAT1* in transgenic plants. (**F**) The expression patterns of *Fd-GOGAT1* in different tissues as analyzed by qRT-PCR. (**G**) The Fd-GOGAT activity in wild-type, *lc7* and complemented *lc7* plants. (**H**) The phenotypes of T_1_ transgenic plants and their parents at the booting stage. (**I**) The lesion length of *lc7* mutant and complemented *lc7* plants 14 days after inoculation with *Xoo* strain *PXO99*. Nip-V and *lc7*-V are transgenic plants from the Nipponbare and *lc7* mutant transformed with the pCAMBIA1301 vector, respectively; *lc7*-C is the function-complemented *lc7* plant; and NRi is the *OsFd-GOGAT1* RNAi plant. The scale bar is indicated.

The genetic analysis of the *lc7* mutant was carried out by crossing with Nipponbare (the parent, *Japonica*), Zhefu 802 (*Indica*) or Minghui 63 (*Indica*). The similar phenotypes of leaf color and disease resistance between the F_1_ plants from these crosses and the wild-type indicate that the yellow and brown-streak leaf trait is controlled by a pair of homozygous recessive genes in the *lc7* mutant. Subsequently, the two derived F_2_ populations, including 2,575 and 23,461 plants, were further investigated. Within these populations, 621 and 5,846 plants exhibited the mutant phenotype, respectively. The segregation ratios also meet Mendel’s laws of inheritance as a pair of homozygous recessive genes. Then, we used map-based cloning to isolate the *lc7* gene. The *lc7* locus was mapped primarily between two simple sequence repeat (SSR) markers, S7 and S4, on chromosome 7. This locus was further narrowed to a 78-kb region between S16 and S22 with sequence-tagged site markers (STS) (Fig. 5A). Nine ORFs were predicted in the 78-kb region (candidate genes are listed in Supplemental Table S1). After sequence analysis of the 10 candidate genes in the *lc7* mutant, we found that the *LOC_Os07g46460* gene with a single-nucleotide A-to-G substitution at the 983^rd^ position, causing a change from asparagine (AAC) to serine (AGC), is a potential candidate of *lc7. LOC_Os07g46460* encodes the rice ferredoxin-dependent glutamate synthase1 (*OsFd-GOGAT1*) and has extremely high sequence similarity with Fd-GOGAT in *Arabidopsis thaliana*. To verify the hypothesis, a genetic complementation test was carried out. The full-length cDNA of *OsFd-GOGAT1* driven by its native promoter was introduced into *lc7* plants and generated 60 hygromycin-positive T_0_ transgenic plants (Fig. 5B,D). As expected, the yellow leaf and brown streak phenotype was not observed in most of the positive *lc7*-C transgenic lines. Except for low fertility, other traits of these positive transgenic plants were similar to those of the wild-type (Fig. 5G). In addition, the enzyme activity of Fd-GOGAT1 was analyzed. The results showed that the *lc7* mutant had a lower enzyme activity, while the Nipponbare and complemented transgenic plants had a higher enzyme activity (Fig. 5F). The disease resistance of these positive transgenic plants was also analyzed with *Xoo* race PXO99. Two weeks after inoculation, the disease resistance was surveyed. The average lesion lengths were 6.2 cm, 0.3 cm and 5.0 cm in the wild-type, *lc7* and *lc7* complemented transgenic plants (Fig. 5H). Similarly, the *OsFd-GOGAT1* RNAi silence binary plasmid was constructed, and transferred into the rice variety Nipponbare to yield 15 hygromycin-positive independent T_0_ lines (Fig. 5C,E). Ten of these lines showed a similar phenotype to that of the *lc7* mutant (Fig. 5G,H). In addition, the full-length cDNA of *Lc7* driven by the 35S promoter was transferred into Nipponbare, and 18 hygromycin-positive T_1_ plants overexpressing *Lc7* were obtained. No obvious difference was observed in the growth or morphology among the control and *Lc7*-overexpressing plants under normal growth conditions; similarly, there was no different susceptibility phenotype between these transgenic plants and wild-type plants (data not shown). Together, these results indicate that Fd-GOGAT1 is LC7 and that the amino acid change of OsFd-GOGAT1 causes the phenotype of the *lc7* mutant. qRT-PCR revealed that the expression of *OsFd-GOGAT1* varied in the roots and leaves and is especially high in the leaves (Fig. 5F).

### Differential gene expression analysis of the *lc7* mutant

To study the influence of *lc7* on disease resistance and nitrogen metabolism, a digital expression profiling sequencing analysis was carried out. A total of 51,279 transcripts were analyzed to check for differences between *lc7* and Nipponbare plants 8 hours after being inoculated with or without *Xoo* race PXO99. Differentially expressed genes (DEGs) were detected according to the conventional criteria (2-fold cutoff, p-values <0.05). A total of 684 and 1,121 genes were up-regulated, respectively, in the *lc7* mutant compared to the wild-type plant after H_2_O treatment and bacterial inoculation. Likewise, 622 and 838 genes were down-regulated, respectively. Most of these DEGs were further verified by real-time quantitative PCR; the qRT-PCR primer sequences are listed in Supplemental Table S2. A functional cluster analysis of the differentially expressed genes revealed that the genes that were involved in nitrogen absorption, ammonium assimilation and defense were greatly activated in the *lc7* plants. For example, *PAL*, *GST*, *PRs*, *NPR1*, nitrate reductase, asparagine synthetase, glutathione S-transferase, and ferric reductase genes and dozens of WRKY transcription factor genes were significantly induced in the *lc7* mutant (Table 2).

### The substitution of amino acids in *lc7* results in its conformational change

Glutamate synthase (GOGAT) can be divided into two groups: ferredoxin-dependent glutamate synthase and NADH-dependent glutamate synthase. Fd-GOGAT is found in all plants, and its amino acid sequence is conserved. Specifically, OsFd-GOGAT1 is highly homologous to ZmFd-GOGAT1, as determined through an alignment analysis of sequences from different plants (Fig. S1A,C). The large (alpha, GltB) subunit of this protein consists of three domains: N-terminal domain (aminotransferase domain), central domain (FMN-binding domain), and C-terminal domain. Compared to the amino acid sequence of wild-type, the mutation site is located in the conserved glutamine amidotransferases class-II domain in *lc7* mutant. Though asparagine and serine are both neutral polar, the polar group of serine is a hydroxyl and finally results in the conformational or functional change of glutamine amidotransferase domain (http://swissmodel.expasy.org/; Fig. S1A,B). Previous study showed that there are six steps in the glutamate synthase reaction. The transfer of the ammonia molecule from the glutaminase site to the synthase site through a 30 Å long intramolecular tunnel is catalyzed by the glutamine amidotransferase with certain conformation^22^. The mutation may greatly influence the ammonia molecular transfer.

## Discussion

Many leaf color mutants have been reported in plants, including in *Zea mays*^23^, *Pisum sativum*^24^, *Nicotiana tabacum*^25^, *Arabidopsis thaliana*^26^ and *Oryza sativa*^27^. All of these mutants are characterized as having chlorophyll-deficient mutations, and most of them exhibit an albino, etiolated or green-white-stripe phenotype. Here, we characterized a defective Fd-GOGAT enzyme in the rice *lc7* mutant, in which chlorophyll synthesis is dramatically blocked and anti-oxidant defense is activated, resulting in the activation of broad-spectrum bacterial blight resistance. These results indicate that glutamate synthase plays an important role in photosynthesis, chlorophyll biosynthesis and defense response, in addition to ammonium assimilation.

Nitrogen (N) not only is the most important nutrient for plants, but also is a major component of chlorophyll. Nitrogen deficiency can cause leaves chlorosis due to decreased levels of chlorophyll^28^. Nitrogen-deficient plants tend to be stunted, grow slowly, and produce fewer tillers than normal. N-deficient crops may reach maturity earlier than do plants with adequate nitrogen. In contrast, nitrogen-excessive plants experience increased susceptibility to disease and insect attacks. Extremely high levels of nitrogen may actually result in decreased yield, and excess nitrogen may negatively affect seed quality. Thus, the use of nitrogen in equilibrium is very important. Among the many enzymes in nitrogen metabolism, Fd-GOGAT is a key enzyme that converts the ammonium that is generated from nitrogen metabolism or photorespiration. Plants that are deficient in Fd-GOGAT activity are significantly affected in photosynthesis, the photorespiratory cycle and chlorophyll biosynthesis and have been described in *A. thaliana*, such as *gluS*^29^, *gltS*^19^, *gls*^18^, and *glu1*^30^, and in other species, such as barley^31^ and tobacco^32^. Mutants that are defective in Fd-GOGAT show reversible yellow phenotypes and adverse agronomic traits, resulting in decreased yield^33^, which agrees with our results. The *lc7* mutant phenotype of yellow leaves with brown streaks after the four-leaf stage indicates that the *lc7* gene may be involved in photosynthesis and chlorophyll biosynthesis. The *lc7* gene encodes a mutant Fd-GOGAT1 protein as determined by map-based cloning. There is a single-nucleotide A-to-G substitution at the 983^rd^ position that causes an asparagine-to-serine change in the aminotransferase domain. The conversion of ammonium that is generated from nitrate assimilation or photorespiration into glutamate and other amino acids is controlled sequentially by glutamine synthetase and glutamate synthase. The conformation change of OsFd-GOGAT1 may result in a lower aminotransferase activity and lower glutamate synthesis and ammonia accumulation. Excess ammonia has toxic effects on cells by quickly passing through the biomembrane to stimulate the production of excessive levels of ROS molecules. However, it is worth noting that the presence of excess ROS did not lead to cell death. Although the mechanisms of ROS are extremely complex and controversial, there are two possible explanations for this phenomenon. One explanation is that the change from asparagine to serine in the *lc7* aminotransferase domain does not result in a significant loss of function and that the elevated ROS concentration is far a destructive level; the other explanation is that *lc7* mutant leaves mainly produce excess H_2_O_2_. Previous reports have suggested the superoxide, but not H_2_O_2_, causes cell death^34^. To prevent the excessive accumulation of ROS from reaching destructive levels and to maintain cellular redox balance, ROS increases the activity of some ROS-scavenging enzymes, such as Glutathione S-transferase, SOD, MDA, CAT, and GST. In addition, ROS can act as transmissible signal to induce the expression of defense-related genes, such as *MAPK*, *PR-1*, *GST* and *GPx*, resulting in a systemic defense response^35^,^36^,^37^.

The digital expression profiling sequencing analysis shows that many defense pathways that are involved in broad spectrum resistance are activated in the *lc7* mutant. In addition to the genes that are involved in detoxification mechanisms, the expressions of numerous stress-related genes, such as WRKY, P450 and NBS, were affected in the *lc7* mutant (Table 2). WRKY proteins are a large family of transcription factors that mainly participate in plant biotic stress responses. So far, more than one hundred *WRKY* genes have been predicted in the rice genome, and most of the genes that are involved in the defense response have been characterized: *OsWRKY6*^38^, *OsWRKY13*^39^, *OsWRKY22*^40^, *OsWRKY30*^41^, *OsWRKY31*^42^, *OsWRKY33*^43^, *OsWRKY45*^44^, *OsWRKY53*^45^, *OsWRKY62*^46^, *OsWRKY71*^47^, *OsWRKY76*^48^, *OsWRKY77*^49^, and *OsWRKY89*^50^. In the *lc7* mutant, *OsWRKY19*, *OsWRKY23*, *OsWRKY47*, *OsWRKY65*, *OsWRKY70* and *OsWRKY79* are up-regulated, while *OsWRKY68* is down-regulated. Thus, *lc7* participates in broad-spectrum resistance to *Xoo*, possibly being involved in three ways. The first way is that excessive ammonia results in the significant production of active oxygen species; the second way is that the decreased glutamate supply will cause a shortage of some nutrients for *Xoo* growth in host or some regulatory factors for the activation/suppress of susceptibility/resistance genes; and the third way is that a molecular signal, such as ROS or SA, triggers the inner immune system to confer upon *lc7* systemic acquired resistance (SAR).

In brief, chlorophyll synthesis, nitrogen metabolism and disease resistance are the three most important factors affecting rice production. OsFd-GOGAT1 is involved in the three pathways, indicating that it plays a key role in the regulation of three important agriculture traits in rice production. The functional study of OsFd-GOGAT1 will lead to the development of new high-yielding varieties.

## Materials and Methods

### Plant materials and growth conditions

The rice (*Oryza sativa* L.) *lc7* mutant was isolated from Co^21^-radiated Nipponbare (ssp japonica). Most of the plants were cultivated in the experimental field of the Institute of Genetics and Developmental Biology (IGDB) in Beijing. An F_2_ mapping population was constructed by crossing *lc7* with Minghui 63 (ssp. indica). The other plants that were used for the treatment were grown under greenhouse conditions (25 °C, 60–80% relative humidity) to the five-leaf stage.

### Transmission electron microscopy analysis

The wild-type and *lc7* mutant leaf samples were collected from 6-week-old seedling and mature plants. All of the plants were grown under a controlled environment with the same light intensity, temperature and living conditions. First, the leaves were cut to approximately 5 mm in length and fixed in a solution of 2% glutaraldehyde. Next, these samples were further fixed in a solution of 1% OsO_4_, stained with uranyl acetate and dehydrated in an ethanol series. These samples were embedded in Spurr’s medium, sliced to 50 nm in thickness, and stained again. Then, these slices were examined using aJEM-1230 electron microscope (JEOL, Tokyo, Japan) at the voltage of 80 kV.

### Histochemical Detection

Because the leaves of the *lc7* mutant exhibited the yellow-leaf phenotype three weeks after sowing, leaf samples were harvested for histochemical analysis after the yellow-leaf phenotype appeared. ROS accumulation was detected as described previously with some modifications. H_2_O_2_ was detected by DAB staining as described previously with some modification^51^. The fully expanded leaves of 2-month-old wild-type and *lc7* plants were detached and infiltrated with a 0.1% DAB solution. The sampled leaves were placed in a growth chamber for 5 hours at 28 °C and placed in boiling ethanol (95%) for 10 min. The chlorophyll was removed by incubating in 95% (v/v) ethanol overnight before photographing. Superoxide (O_2_^−^) accumulation in rice leaves was visualized by 0.1% NBT staining as previously described^52^. The fully expanded leaves of 2-month-old wild-type and *lc7* plants were stained with NBT solution. After staining overnight, the chlorophyll was removed by incubating in 95% (v/v) ethanol overnight. Dead cells were stained using detached leaves^53^. Leaves of 2-month-old wild-type and *lc7* plants were submerged in trypan blue solution at 70 °C for 10 min and then heated in boiling water for 2 min and left to stain overnight. After destaining in chloral hydrate solution (25 g of chloral hydrate in 10 ml of H_2_O) for 3 days, the samples were equilibrated with 70% glycerol for microphotography.

### Preparation of tissue extracts

Individuals were also dissected on a board to remove the gills and digestive gland. After dissection, the tissues from each specimen were immediately placed on ice and frozen at −70 °C. In the laboratory, the tissue samples were homogenized in ten volumes (w/v) of 100 mM Tris-HCl buffer (pH 7.5). Each homogenate was sonicated briefly (2–3 s) using an ultrasonic processor and centrifuged at 9,000 g at 4 °C for 15 min^54^. After centrifugation, the supernatants were collected and immediately used for the biochemical analyses. All of the assays were performed in duplicate.

### Biochemical assays

Malondialdehyde (MDA) is a marker of lipid peroxidation^55^ and was determined with an MDA assay kit (Beyotime, China) with some modifications. Briefly, leaves (0.02 g fresh weight) were homogenized in 2 ml of phosphate buffer (pH 7.8) containing 1% PVP and centrifuged at 2,500 *g* for 10 min. The concentration of MDA in the supernatant was determined according to the reaction with thiobarbituric acid, and the absorbance was measured at 450, 532, and 600 nm. According to the formula 6.45 × (OD532–OD600) − 0.56 × OD450, the MDA content was expressed as micromolar of MDA per gram fresh weight.

The thioredoxin reductase (TR) activity was measured with an endpoint method using a thioredoxin-coupled insulin reduction assay^56^. The CAT activity was measured based on the decomposition of H_2_O_2_^57^. The SOD activity was determined by the degree of inhibition in the reduction of cytochrome c by the superoxide anion that was generated by xanthine oxidase/hypoxanthine following the method described by McCord and Fridovich^58^. The glutathione S-transferase (GST) activity was measured by the method of Habig using reduced glutathione (GSH) and 1-chloro-2, 4-dinitrobenzene (CDNB) as substrates^59^. The enzymatic activities and MDA concentration were determined with a Shimadzu UV-2100 spectrophotometer at 37 °C. The MDA concentration and all of the enzyme activities were measured in duplicate for each sample. The total protein content was determined by a colorimetric method (Bio-Rad Protein Assay) using bovine serum albumin (BSA) as a standard to normalize all of the biochemical results.

### Enzyme assays

For the enzyme assay of Fd-GOGAT^60^, 200 mg of cotyledon was homogenized on ice with a mortar and pestle using 2 ml of extraction buffer (50 mM HEPES/KOH pH 7.5, 0.5 mM ethylenediaminetetraacetic acid (EDTA), 100 mM KCl, 0.5% (v/v) TritonX 100, 0.1% (v/v) mercaptoethanol, and 1 mM PMSF (added just before extraction)) in the presence of 1 g of quartz sand and 0.3 g of Dowex 1, 1 × 2–400 (equilibrated with extraction buffer). After the addition of another 4 ml of extraction buffer, the homogenate was clarified by centrifugation (20 min, 39,000 g), and the supernatant was used for the enzyme assay. The enzyme activity Fd-GOGAT was measured by determining the formation of glutamate in the reaction between oxoglutarate and glutamine. The reaction mixture (final volume 1.5 ml) consisted of 100 μl of 100 mM glutamine in 50 mM HEPES/KOH, pH 7.5; 100 μl of 100 mM oxoglutarate in 50 mM HEPES/KOH, pH 7.5; 100 μl of 150 mM methylviologen; and 400 μl of crude extract.

After preincubation at 30 °C, the reaction was started by the addition of 100 μl of reductant (47 mg Na_2_S_2_O_4_, 50 mg of NaHCO_3_ dissolved in 1 ml of distilled water). After a 20-min incubation at 30 °C, the reaction was terminated by adding 1 ml of ethanol. This mixture was transferred completely to a Dowex-acetate column (1 × 8, 200–400 mesh size, 12-mm QS, 35-mm length). Glutamine was eluted from the column with 15 ml of distilled water. To remove residual water, the column was centrifuged for 2 min at 1500 rpm. Glutamate was then eluted from the column with 5 ml of 3 M acetic acid, and the centrifugation step was repeated. An aliquot of 5 ml of acetic acid was found to be sufficient to elute more than 95% of the glutamate. The concentration of glutamate in the eluate was determined by a ninhydrin assay. An aliquot of 500 μl of the eluate was added to 1 ml of a ninhydrin solution (0.4 g of ninhydrin, 80 ml of 95% ethanol, 1 g of CdCl_2_, 10 ml of acetic acid, and 20 ml of H_2_O). After 10 min of incubation at 80 °C in a water bath, the samples were cooled, and the absorbance was measured at 506 nm. To note any side reactions, control samples were tested, from which oxoglutarate, glutamine or methylviologen were omitted. One unit of enzyme activity represents 1 μmol of glutamate formed/min at 30 °C, The protein was measured by spectrophotometry at 595 nm.

### Map-based cloning of *lc7*

For the map-based cloning of *lc7*, an F_2_ mapping population was constructed by crossing *lc7* with the Indica cultivar Minghui 63. The *lc7* locus was first mapped between markers S7 and S4 of chromosome 7 using 621 F_2_ mutant plants, and the targeted gene was further narrowed to 78-kb region using markers S16 and S22 and 5,846 F_2_ mutant plants. Nine predicted ORFs exist within this interval (Supplemental Table S1). Sequencing analysis revealed that *LOCOs07g046460* containing a single-base substitution is the most probable candidate gene of *lc7*.

### Complementation of *lc7* vector construction

For the complementation of *lc7*, the 4,848-bp cDNA fragment of *LC7* (*OsFd-GOGAT*) driven by its native promoter was obtained by digestion with *XhoI* and *SacI* and subsequently ligated into binary vector pCambia 1301. The plasmid was introduced into Agrobacterium tumefaciens AGL-1 by freeze-thaw transformation. Then, the mutant *lc7* callus was transformed by an Agrobacterium-mediated method as described previously^61^. The full-length cDNA of amplified *OsFd-GOGAT* was digested with *Sac*I and *Sal*I and subsequently ligated into the pXQAct^21^, resulting in p*OsFd-GOGAT*-O vector. The primer sequences that were used for vector construction are listed in Supplemental Table S3.

### Quantitative RT-PCR analysis

Total RNA was extracted from the leaves of rice plants using a TRIzol Kit according to the user’s manual (Invitrogen, CA, USA). Three micrograms of total RNAs were treated with DNase I and used for cDNA synthesis with M-MLV Reverse Transcriptase (Promega, Madison, WI, USA). Real-time PCR experiments were performed using gene-specific primers and SYBR Green I Mix with the CFX96 Real-Time PCR Detection System (Bio-Rad, USA). Rice Actin1 was used as an internal control.

### Bacterial inoculation

To evaluate bacterial blight disease resistance, plants were inoculated with the *Xanthomonas oryzae* pv. *Oryzae* strain at the tillering stage by the leaf-clipping method^60^. The disease was scored by measuring the lesion length two weeks after inoculation. Ninety leaves obtained from 30 plants (three leaves per plant) were inoculated, measured and subjected to statistical analysis. Three independent replicates were performed.

## Additional Information

**How to cite this article**: Chen, H. *et al.* The Fd-GOGAT1 mutant gene *lc7* confers resistance to *Xanthomonas oryzae* pv. *Oryzae* in rice. *Sci. Rep.* **6**, 26411; doi: 10.1038/srep26411 (2016).

## Supplementary Material

Supplementary Information

Supplementary Dataset 1-3

## Figures and Tables

**Table 1:** Virulence assays of Nipponbare, *lc7* and *lc7*-C from seven strains of *Xoo*.

*Xoo* strain	Lesion length (cm)		
	Nipponbare	*lc7*	*lc7*-C
PXO86	13.9 ± 1.8	1.4 ± 0.3	12.9 ± 1.6
PXO71	13.2 ± 1.7	1.5 ± 0.4	8.2 ± 1.6
PXO79	14.5 ± 1.6	1.7 ± 0.4	13.5 ± 1.5
PXO99	15.3 ± 1.3	1.7 ± 0.7	12.3 ± 1.7
PXO145	13.5 ± 1.9	1.8 ± 0.5	11.3 ± 1.6
PXO280	15.4 ± 1.2	1.6 ± 0.4	16.7 ± 1.3
PXO339	12.3 ± 1.6	1.5 ± 0.7	13.0 ± 1.4

**Table 2:** Selected differentially expressed genes that were functionally classified in the *lc7* mutant compared to the wild-type by microarray analysis.

Accession number	Functional categories	Fold change (*lc7*/WT)			
		0 hours (DEG)	8 hours (DEG)	0 hours (qPCR)	8 hours (qPCR)
Ferredoxin–relative protein					
LOC_Os05g04120	Ferroportin	8.5	7.9	5.4	3.7
LOC_Os08g35210	Ferric reductase	−1.0	–	−0.2	2.3
LOC_Os09g26660	Ferric reductase	−1.5	–	−0.3	1.8
LOC_Os07g46460	Ferredoxin-dependent glutamate synthase	1.0	–	0.6	0.8
Glutathione S-transferase					
LOC_Os01g27340	Glutathione S-transferase	10.6	7.9	5.3	5.9
LOC_Os10g34020	Glutathione S-transferase	8.1	–	–	–
LOC_Os03g04250	Glutathione S-transferase	7.8	–	–	–
LOC_Os10g38590	Glutathione S-transferase	4.2	–	–	–
Pathogenesis and disease resistance related					
LOC_Os03g20840	Disease resistance RPP13-like protein	8.9	–	2.4	4.7
LOC_Os01g08370	Symbiosis-related disease resistance protein	−1.3	−	0.4	2.2
LOC_Os01g65920	F-box/LRR-repeat protein	1.2	–	0.1	0.7
LOC_Os12g13550	NBS-LRR disease resistance protein	−7.8	–	0.3	−1.7
LOC_Os05g34550	MLO domain containing protein	−1.1	–	–	–
LOC_Os11g41210	Disease resistance protein RPM1	2.2	–	–	–
LOC_Os11g37700	Pleiotropic drug resistance protein	3.7	–	–	–
LOC_Os01g06900	Verticillium wilt disease resistance protein Ve2	−8.0	–	–	–
LOC_Os06g51050	CHIT7 - Chitinase family protein precursor	4.6	–	–	–
Transcription factor					
LOC_Os03g21710	OsWRKY79	4.8	–	4.6	7.0
LOC_Os03g23270	OsWRKY23	7.9	–	1.1	2.6
LOC_Os04g51560	OsWRKY68	−1.8	–	−0.3	0.7
LOC_Os07g48260	OsWRKY47	1.3	–	0.8	1.9
LOC_Os12g02470	OsWRKY65	8.4	–	2.1	0.6
LOC_Os05g49620	OsWRKY19	8.2	–	2.4	4.5
LOC_Os05g39720	OsWRKY70	1.9	–	0.8	–
LOC_Os03g32230	ZOS3-12 - C2H2 zinc finger protein	8.3	–	3.4	–
LOC_Os05g02390	ZOS5-02 - C2H2 zinc finger protein	8.1	–	4.3	–
LOC_Os07g22730	AP2 domain containing protein	9.2	–	–	–
LOC_Os02g49480	Helix-loop-helix D-binding protein	3.3	–	–	–
Nitrogen absorption and ammonium assimilation					
LOC_Os02g53130	Nitrate reductase	−7.9	–	−1.7	−1.2
LOC_Os02g02210	Aminotransferase	11.2	–	8.6	–
LOC_Os03g18130	Asparagine synthetase	2.6	–	3.4	–
LOC_Os08g02030	Transferase family protein	10.9	–	4.3	–
LOC_Os04g38680	Transmembrane amino acid transporter protein	9.6	–	2.1	–
P450 family protein					
LOC_Os03g40540	Cytochrome P450	−1.4	–	−0.7	0.4
LOC_Os11g18570	Cytochrome P450	1.6	–	1.0	–
LOC_Os10g30390	Cytochrome P450	8.0	–	–	–
LOC_Os06g19070	Cytochrome P450	8.1	–	–	–
LOC_Os07g44140	Cytochrome P450	3.2	–	–	–
LOC_Os02g36190	Cytochrome P450	2.9		–	–
Redoxreaction					
LOC_Os11g25160	Tropinone reductase 2	1.14	–	0.1	–
LOC_Os01g01660	Isoflavone reductase	−1.9	–	1.5	–
LOC_Os07g02440	Peroxidase precursor	1.7	–	0.8	–
LOC_Os04g41260	Amine oxidase	15.6	–	3.3	–
LOC_Os05g10930	OsGrx_C15 - glutaredoxin subgroup III	−1.2	−1.2	−1.0	1.1
LOC_Os04g59200	Peroxidase precursor	9.3	–	–	–
Abiotic stress					
LOC_Os12g43380	Thaumatin	2.4	–	1.3	–
LOC_Os10g35070	Alpha-galactosidase precursor	4.2	–	3.3	–
Hormone					
LOC_Os02g06910	Auxin response factor 6	−1.1	–	–	–
LOC_Os10g34230	Cytokinin dehydroge-se precursor	8.1	–	–	–
LOC_Os03g38600	Secretory carrier-associated membrane protein	2.6	–	–	–
LOC_Os02g51930	cytokinin-O-glucosyltransferase 2	4.3	–	–	–
Secondary metabolism					
LOC_Os02g36140	Terpene synthase	10.2	–	–	–
LOC_Os01g64910	Anthocyanidin 5,3-O-glucosyltransferase	9.8	–	–	–
LOC_Os11g42200	Laccase precursor protein	9.4	–	–	–
LOC_Os04g56030	Glycine-rich cell wall structural protein precursor	8.3	–	–	–
Others					
LOC_Os01g71670	Glycosyl hydrolases family 17	2.0	–	3.1	–
LOC_Os06g43630	Sucrose-phosphate synthase	−1.1	–	–	–
LOC_Os01g24710	Jacalin-like lectin domain containing protein	8.0	–	1.7	–
LOC_Os02g35329	RING-H2 finger protein ATL3F	4.3	–	4.4	–
LOC_Os04g21130	F-box protein PP2-B1	9.8	–	3.4	–
LOC_Os07g43240	SKP1-like protein 1B	8.6	–	3.8	–
LOC_Os08g31140	Heavy metal-associated domain containing protein	−3.4	–	−1.3	–
LOC_Os06g35060	Heavy metal-associated domain containing protein	10.4	–	–	–
LOC_Os02g16940	OsSub13 - Putative Subtilisin homologue	8.8	–	–	–
LOC_Os09g34250	UDP-glucoronosyl and UDP-glucosyl transferase domain containing protein	13.8	–	–	–
LOC_Os10g29650	Retrotransposon protein	2.5	–	1.1	–
LOC_Os04g45920	Protein kinase domain containing protein	0.4	–	1.1	–
LOC_Os01g06310	Glycine-rich cell wall structural protein precursor	−2.1	–	−1.3	–
LOC_Os01g01660	Isoflavone reductase	−1.9	–	0.4	1.5
LOC_Os01g01880	Expressed protein	–	–	−0.7	−3.3
LOC_Os01g06310	Glycine-rich cell wall structural protein precursor	4.1	–	−1.0	2.1
LOC_Os01g34970	MDR-like ABC transporter	–	–	0.0	−0.3
LOC_Os01g48360	Expressed protein	−1.1	–	0.1	2.1
LOC_Os01g71670	Glycosyl hydrolases family 17	–	–	0.0	3.1
LOC_Os02g42330	nitrilase	1.5	–	0.3	0.4
LOC_Os03g18130	Asparagine synthetase	2.6	3.4	3.5	6.7
LOC_Os04g21230	Glycosyl hydrolases family 17	–	–	0.8	−0.3
LOC_Os04g45920	Protein kinase domain containing protein	–	–	1.1	2.6
LOC_Os07g01340	Gibberellin 2-beta-dioxyge-se 7	–	2.5	0.1	0.1
LOC_Os07g02440	Peroxidase precursor	1.7	−2.4	0.1	−1.3
LOC_Os07g03730	SCP-like extracellular protein	8.4	4.4	6.1	5.5
LOC_Os08g31140	Heavy metal-associated domain containing protein	–	5.0	0.8	3.1
LOC_Os10g29650	Retrotransposon protein	2.5	−1.4	0.3	1.1
LOC_Os10g35070	Alpha-galactosidase precursor	–	9.9	−0.2	2.1
LOC_Os11g25160	Tropinone reductase 2	1.1	−1.4	−0.2	1.2
LOC_Os11g37970	WIP5 - Wound-induced protein	–	4.6	0.9	4.0
LOC_Os12g43380	Thaumatin	2.4	−1.0	3.7	2.8
LOC_Os01g01880	Expressed protein	–	–	−3.3	−0.3
LOC_Os01g48360	Expressed protein	−1.1	1.4	−1.3	2.6

## References

[b1] Gnanamanickam S. S., Priyadarisini V. B., Narayanan N. N., Vasudevan P. & Kavitha S. An overview of bacterial blight disease of rice and strategies for its management. Curr. Sci. India 77, 1435–1444 (1999).

[b2] Verdier V. *et al.* Transcription activator-like (TAL) effectors targeting OsSWEET genes enhance virulence on diverse rice (*Oryza sativa*) varieties when expressed individually in a TAL effector-deficient strain of *Xanthomonas* oryzae. New Phytol. 196, 1197–1207, 10.1111/j.1469-8137.2012.04367.x (2012). 23078195

[b3] Bhasin H. *et al.* New PCR-based sequence-tagged site marker for bacterial blight resistance gene *Xa38* of rice. Mol. Breeding 30, 607–611, 10.1007/s11032-011-9646-y (2012).

[b4] Song W. Y. *et al.* A receptor kinase-like protein encoded by the rice disease resistance gene, Xa21. Science 270, 1804–1806 (1995). 8525370 10.1126/science.270.5243.1804

[b5] Yoshimura S. *et al.* Expression of Xa1, a bacterial blight-resistance gene in rice, is induced by bacterial inoculation. Proc. Natl. Acad. Sci. USA 95, 1663–1668 (1998). 9465073 10.1073/pnas.95.4.1663 PMC19140

[b6] Sun X. *et al.* *Xa26*, a gene conferring resistance to *Xanthomonas oryzae* pv. *oryzae* in rice, encodes an LRR receptor kinase-like protein. Plant J. 37, 517–527 (2004). 14756760 10.1046/j.1365-313x.2003.01976.x

[b7] Jiang G. H. *et al.* Testifying the rice bacterial blight resistance gene *xa5* by genetic complementation and further analyzing xa5 (Xa5) in comparison with its homolog TFIIAgamma1. Mol. Genet. Genomics 275, 354–366, 10.1007/s00438-005-0091-7 (2006). 16614777

[b8] Iyer A. S. & McCouch S. R. The rice bacterial blight resistance gene *xa5* encodes a novel form of disease resistance. Mol. Plant Microbe. Interact. 17, 1348–1354, 10.1094/MPMI.2004.17.12.1348 (2004). 15597740

[b9] Chu Z. *et al.* Targeting *xa13*, a recessive gene for bacterial blight resistance in rice. Theor. Appl. Genet. 112, 455–461, 10.1007/s00122-005-0145-6 (2006). 16328230

[b10] Wu L., Goh M. L., Sreekala C. & Yin Z. XA27 depends on an amino-terminal signal-anchor-like sequence to localize to the apoplast for resistance to *Xanthomonas oryzae* pv *oryzae*. Plant Physiol 148, 1497–1509, 10.1104/pp.108.123356 (2008). 18784285 PMC2577279

[b11] Tian D. *et al.* The rice TAL effector-dependent resistance protein XA10 triggers cell death and calcium depletion in the endoplasmic reticulum. Plant Cell 26, 497–515, 10.1105/tpc.113.119255 (2014). 24488961 PMC3963592

[b12] Wang C. *et al.* High-resolution genetic mapping of rice bacterial blight resistance gene *Xa23*. Mol. Genet. Genomics 289, 745–753, 10.1007/s00438-014-0848-y (2014). 24715026

[b13] Kottapalli K. R., Lakshmi Narasu M. & Jena K. K. Effective strategy for pyramiding three bacterial blight resistance genes into fine grain rice cultivar, *Samba Mahsuri*, using sequence tagged site markers. Biotechnol. Letters 32, 989–996, 10.1007/s10529-010-0249-1 (2010). 20349335

[b14] Forde B. G. & Lea P. J. Glutamate in plants: metabolism, regulation, and signalling. J. Exp. Bot. 58, 2339–2358, 10.1093/jxb/erm121 (2007). 17578865

[b15] Kissen R. *et al.* Transcriptional profiling of an *Fd-GOGAT1*/*GLU1* mutant in *Arabidopsis thaliana* reveals a multiple stress response and extensive reprogramming of the transcriptome. BMC Genomics 11, 190, 10.1186/1471-2164-11-190 (2010). 20307264 PMC2858750

[b16] Botella J. R., Verbelen J. P. & Valpuesta V. Immunocytolocalization of ferredoxin-GOGAT in the cells of green leaves and cotyledons of lycopersicon esculentum. Plant Physiol. 87, 255–257 (1988). 16666114 10.1104/pp.87.1.255 PMC1054735

[b17] Jamai A., Salome P. A., Schilling S. H., Weber A. P. & McClung C. R. *Arabidopsis* photorespiratory serine hydroxymethyltransferase activity requires the mitochondrial accumulation of ferredoxin-dependent glutamate synthase. Plant Cell 21, 595–606, 10.1105/tpc.108.063289 (2009). 19223513 PMC2660619

[b18] Coschigano K. T., Melo-Oliveira R., Lim J. & Coruzzi G. M. *Arabidopsis gls* mutants and distinct Fd-GOGAT genes. Implications for photorespiration and primary nitrogen assimilation. Plant Cell 10, 741–752 (1998). 9596633 10.1105/tpc.10.5.741 PMC144371

[b19] Suzuki A. & Rothstein S. Structure and regulation of ferredoxin-dependent glutamase synthase from *Arabidopsis thaliana*. Cloning of cDNA expression in different tissues of wild-type and gltS mutant strains, and light induction. Eur. J. Biochem. 243, 708–718 (1997). 9057836 10.1111/j.1432-1033.1997.00708.x

[b20] Hayakawa T. *et al.* Cellular localization of NADH-dependent glutamate-synthase protein in vascular bundles of unexpanded leaf blades and young grains of rice plants. Planta 193, 455–460, 10.1007/BF00201826 (1994).

[b21] Lin A. *et al.* Nitric oxide and protein S-nitrosylation are integral to hydrogen peroxide-induced leaf cell death in rice. Plant Physiol. 158, 451–464, 10.1104/pp.111.184531 (2012). 22106097 PMC3252116

[b22] Mouilleron S. & Golinelli-Pimpaneau B. Conformational changes in ammonia-channeling glutamine amidotransferases. Curr Opin Struct Biol 17, 653–664, 10.1016/j.sbi.2007.09.003 (2007). 17951049

[b23] Sheridan W. F. & Neuffer M. G. Defective kernel kutants of kaize II. korphological and embryo culture studies. Genet. 95, 945–960 (1980). 10.1093/genetics/95.4.945 PMC1214278 17249054

[b24] Platten J. D. *et al.* Cryptochrome 1 contributes to blue-light sensing in pea. Plant Physiol. 139, 1472–1482, 10.1104/pp.105.067462 (2005). 16244154 PMC1283782

[b25] Koivuniemi P. J., Tolbert N. E. & Carlson P. S. Characterization of the thylakoid membranes of the tobacco aurea mutant *Su*/*su* and of three green revertant plants. Planta 151, 40–47, 10.1007/BF00384235 (1981). 24301668

[b26] Park S. Y. *et al.* The senescence-induced staygreen protein regulates chlorophyll degradation. Plant Cell 19, 1649–1664, 10.1105/tpc.106.044891 (2007). 17513504 PMC1913741

[b27] Delmas F. *et al.* ABI3 controls embryo degreening through Mendel’s I locus. Proc. Natl. Acad. Sci. USA. 110, E3888–3894, 10.1073/pnas.1308114110 (2013). 24043799 PMC3791760

[b28] Shi R. *et al.* Senescence-induced iron mobilization in source leaves of barley (*Hordeum vulgare*) plants. New Phytol. 195, 372–383, 10.1111/j.1469-8137.2012.04165.x (2012). 22591276

[b29] Somerville C. R. & Ogren W. L. Inhibition of photosynthesis in *Arabidopsis* mutants lacking leaf glutamate synthase activity. Nature 286, 257–259 (1980).

[b30] Jamai A., Salome P. A., Schilling S. H., Weber A. P. & McClung C. R. *Arabidopsis* photorespiratory serine hydroxymethyltransferase activity requires the mitochondrial accumulation of ferredoxin-dependent glutamate synthase. Plant Cell 21, 595–606, 10.1105/tpc.108.063289 (2009). 19223513 PMC2660619

[b31] Kendall A. C., Wallsgrove R. M., Hall N. P., Turner J. C. & Lea P. J. Carbon and nitrogen metabolism in barley (*Hordeum vulgare* L.) mutants lacking ferredoxin-dependent glutamate synthase. Planta 168, 316–323, 10.1007/BF00392355 (1986). 24232139

[b32] Ferrario-Mery S. *et al.* Modulation of amino acid metabolism in transformed tobacco plants deficient in Fd-GOGAT. Plant Soil 221, 67–79, 10.1023/A:1004715208478 (2000).

[b33] Ferrario-Mery S. *et al.* Diurnal changes in ammonia assimilation in transformed tobacco plants expressing ferredoxin-dependent glutamate synthase mRNA in the antisense orientation. Plant Sci. 163, 59–67, 10.1016/S0168-9452(02)00058-4 (2002).

[b34] Jabs T. Reactive oxygen intermediates as mediators of programmed cell death in plants and animals. Biochem. Pharmacol 57, 231–245 (1999). 9890550 10.1016/s0006-2952(98)00227-5

[b35] Capone R., Tiwari B. S. & Levine A. Rapid transmission of oxidative and nitrosative stress signals from roots to shoots in *Arabidopsis*. Plant Physiol. Biochem. 42, 425–428, 10.1016/j.plaphy.2004.03.005 (2004). 15191746

[b36] Chen Z., Silva H. & Klessig D. F. Active oxygen species in the induction of plant systemic acquired resistance by salicylic acid. Science 262, 1883–1886 (1993). 8266079 10.1126/science.8266079

[b37] Levine A., Tenhaken R., Dixon R. & Lamb C. H_2_O_2_ from the oxidative burst orchestrates the plant hypersensitive disease resistance response. Cell 79, 583–593 (1994). 7954825 10.1016/0092-8674(94)90544-4

[b38] Hwang S. H., Yie S. W. & Hwang D. J. Heterologous expression of *OsWRKY6* gene in *Arabidopsis* activates the expression of defense related genes and enhances resistance to pathogens. Plant Sci. 181, 316–323, 10.1016/j.plantsci.2011.06.007 (2011). 21763543

[b39] Qiu D. *et al.* *OsWRKY13* mediates rice disease resistance by regulating defense-related genes in salicylate- and jasmonate-dependent signaling. Mol. Plant Microbe. Interact. 20, 492–499, 10.1094/MPMI-20-5-0492 (2007). 17506327

[b40] Abbruscato P. *et al.* *OsWRKY22*, a monocot WRKY gene, plays a role in the resistance response to blast. Mol. Plant Pathol. 13, 828–841, 10.1111/j.1364-3703.2012.00795.x (2012). 22443363 PMC6638809

[b41] Shen H. *et al.* OsWRKY30 is activated by MAP kinases to confer drought tolerance in rice. Plant Mol. Biol. 80, 241–253, 10.1007/s11103-012-9941-y (2012). 22875749

[b42] Zhang J., Peng Y. & Guo Z. Constitutive expression of pathogen-inducible OsWRKY31 enhances disease resistance and affects root growth and auxin response in transgenic rice plants. Cell Res. 18, 508–521, 10.1038/cr.2007.104 (2008). 18071364

[b43] Koo S. C. *et al.* OsBWMK1 mediates SA-dependent defense responses by activating the transcription factor OsWRKY33. Biochem. Bioph. Res. Commun. 387, 365–370, 10.1016/j.bbrc.2009.07.026 (2009). 19607808

[b44] Shimono M. *et al.* Rice WRKY45 plays important roles in fungal and bacterial disease resistance. Mol. Plant Pathol. 13, 83–94, 10.1111/j.1364-3703.2011.00732.x (2012). 21726399 PMC6638719

[b45] Chujo T. *et al.* Overexpression of phosphomimic mutated OsWRKY53 leads to enhanced blast resistance in rice. PloS One 9, e98737, 10.1371/journal.pone.0098737 (2014). 24892523 PMC4043820

[b46] Peng Y. *et al.* OsWRKY62 is a negative regulator of basal and Xa21-mediated defense against *Xanthomonas oryzae* pv. *oryzae* in rice. Mol. Plant 1, 446–458, 10.1093/mp/ssn024 (2008). 19825552

[b47] Liu X., Bai X., Wang X. & Chu C. OsWRKY71, a rice transcription factor, is involved in rice defense response. J. Plant Physiol 164, 969–979, 10.1016/j.jplph.2006.07.006 (2007). 16919842

[b48] Yokotani N. *et al.* WRKY76 is a rice transcriptional repressor playing opposite roles in blast disease resistance and cold stress tolerance. J. Exp. Bot. 64, 5085–5097, 10.1093/jxb/ert298 (2013). 24043853 PMC3830488

[b49] Lan A. *et al.* A salicylic acid-induced rice (*Oryza sativa* L.) transcription factor OsWRKY77 is involved in disease resistance of *Arabidopsis thaliana*. Plant Biol. 15, 452–461, 10.1111/j.1438-8677.2012.00664.x (2013). 23061987

[b50] Wang H. *et al.* Overexpression of rice WRKY89 enhances ultraviolet B tolerance and disease resistance in rice plants. Plant Mol. Biol. 65, 799–815, 10.1007/s11103-007-9244-x (2007). 17960484

[b51] Xu Q. *et al.* Comparative proteomic analysis reveals the cross-talk between the responses induced by H_2_O_2_ and by long-term rice black-streaked dwarf virus infection in rice. PloS One 8, e81640, 10.1371/journal.pone.0081640 (2013). 24312331 PMC3842349

[b52] Pandey P., Srivastava R. K. & Dubey R. S. Salicylic acid alleviates aluminum toxicity in rice seedlings better than magnesium and calcium by reducing aluminum uptake, suppressing oxidative damage and increasing antioxidative defense. Ecotoxicology 22, 656–670, 10.1007/s10646-013-1058-9 (2013). 23479061

[b53] Waspi U., Schweizer P. & Dudler R. Syringolin reprograms wheat to undergo hypersensitive cell death in a compatible interaction with powdery mildew. Plant Cell 13, 153–161 (2001). 11158536 PMC102206

[b54] Manduzio H., Monsinjon T., Galap C., Leboulenger F. & Rocher B. Seasonal variations in antioxidant defences in blue mussels Mytilus edulis collected from a polluted area: major contributions in gills of an inducible isoform of Cu/Zn-superoxide dismutase and of glutathione S-transferase. Aquat. Toxicol. 70, 83–93, 10.1016/j.aquatox.2004.07.003 (2004). 15451609

[b55] Janero D. R. Malondialdehyde and thiobarbituric acid-reactivity as diagnostic indices of lipid peroxidation and peroxidative tissue injury. Free Radical Biol.Med. 9, 515–540, 10.1016/0891-5849(90)90131-2 (1990). 2079232

[b56] Yua Q. B. *et al.* AtECB1/MRL7, a thioredoxin-like fold protein with disulfide reductase activity, regulates chloroplast gene expression and chloroplast biogenesis in *Arabidopsis thaliana*. Mol. Plant 7, 206–217, 10.1093/mp/sst092 (2014). 23956074

[b57] Aebi H. Catalase *in vitro*. Method Enzymol. 105, 121–126 (1984). 10.1016/s0076-6879(84)05016-3 6727660

[b58] McCord J. M. & Fridovich I. Superoxide dismutase. An enzymic function for *erythrocuprein* (hemocuprein). J. Biol. Chem. 244, 6049–6055 (1969). 5389100

[b59] Habig W. H., Pabst M. J. & Jakoby W. B. Glutathione S-transferases. The first enzymatic step in mercapturic acid formation. J. Biol. Chem. 249, 7130–7139 (1974). 4436300

[b60] Yang S. Q. *et al.* Molecular and pathogenic characterization of new *Xanthomonas oryzae* pv. *oryzae* strains from the coastline region of Fangchenggang city in China. World J. Microb. Biotechnol. 29, 713–720, 10.1007/s11274-012-1227-7 (2013). 23264131

[b61] Hiei Y. & Komari T. Agrobacterium-mediated transformation of rice using immature embryos or calli induced from mature seed. Nat. Protoc. 3, 824–834, 10.1038/nprot.2008.46 (2008). 18451790

