# Supplementary material for: The Fd-GOGAT1 mutant gene lc7 confers resistance to Xanthomonas oryzae pv. Oryzae in rice
Source: Sci Rep. 2016 May 23;6:26411. doi: 10.1038/srep26411 (PMC4876388; doi:10.1038/srep26411)

The Fd-GOGAT1 mutant gene *lc7* confers resistance to *Xanthomonas oryzae* pv. *Oryzae* in rice

Honglin Chen[1,2,†], Chunrong Li[1,†], Liping Liu[3], Jiying Zhao[1], Xuzhen Cheng[2], Guanghuai Jiang[1,*], Wenxue Zhai[1,*]

[1] Institute of Genetics and Developmental Biology, Chinese Academy of Sciences, Beijing 100101, China
[2] National Key Facility for Crop Gene Resources and Genetic Improvement, Institute of Crop Science, Chinese Academy of Agricultural Sciences, Beijing, 100081, China
[3] State Key Laboratory of Agrobiotechnology, College of Biological Sciences, China Agricultural University, Beijing, 100193, China

[†]These authors contributed equally to this work.

[*]Corresponding author: Guanghuai Jiang or Wenxue Zhai
Tel: +861064807633; Fax: +861064807633
E-mail: ghjiang@genetics.ac.cn or wxzhai@genetics.ac.cn

Supplementary Information

Supplemental Figure S1. Sequence comparison of Fd-GOGAT. (A) Amino acid sequence comparison of OsFd-GOGAT with *Arabidopsis*, maize and barley (the mutation site in the glutamine aminotransferase class-Ⅱ domain, indicated by the arrows). (B) Prediction of 3D structures of lc7 and LC7/OsFd-GOGAT proteins (the mutation site N328S is indicated in red, http://swissmodel.expasy.org). (C) Phylogenetic analysis of GOGAT in plants (indicated by the triangle).

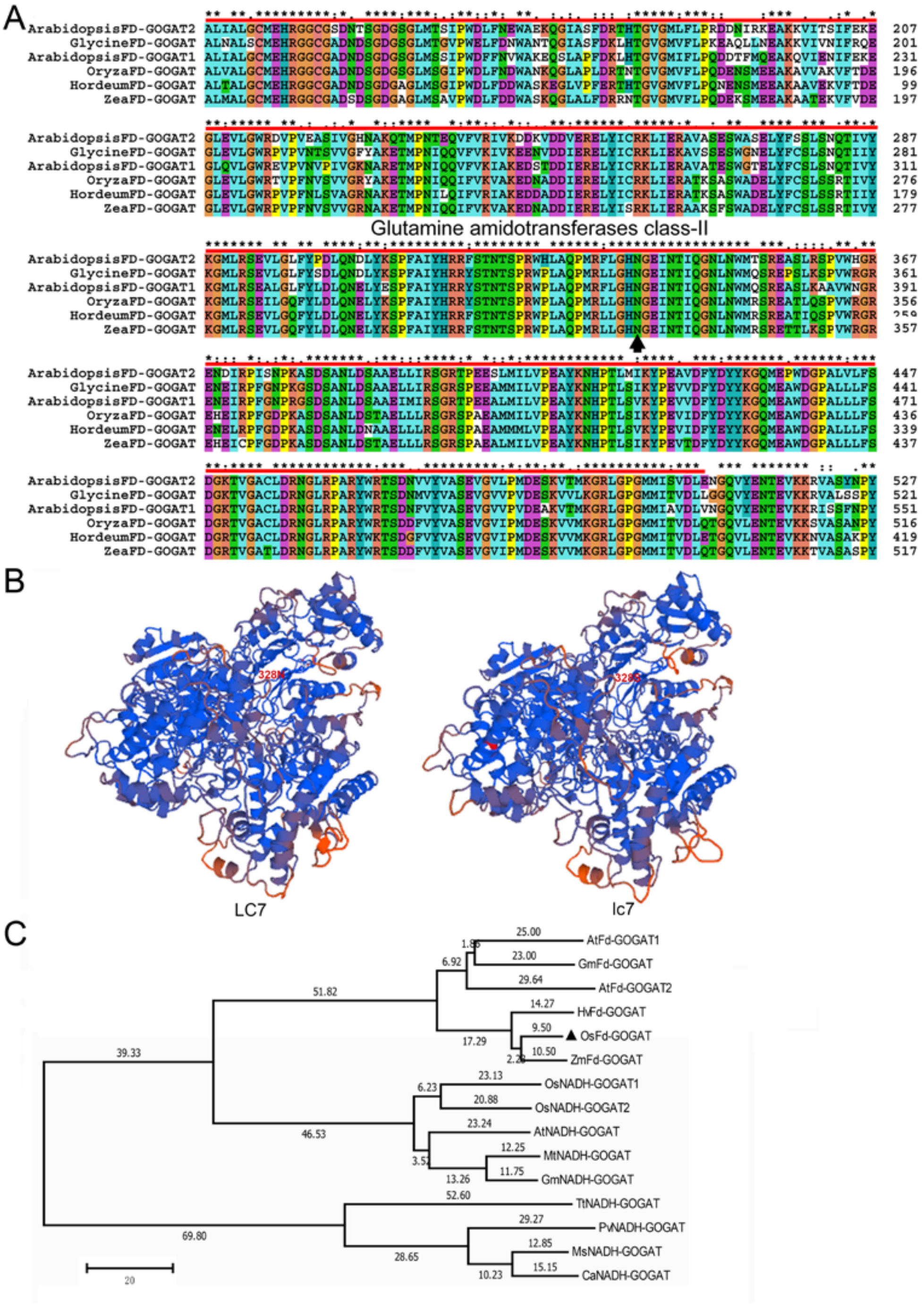

Supplement: Supplementary Information [file srep26411-s1.pdf]
