# Supplementary material for: The Fd-GOGAT1 mutant gene lc7 confers resistance to Xanthomonas oryzae pv. Oryzae in rice
Source: Sci Rep. 2016 May 23;6:26411. doi: 10.1038/srep26411 (PMC4876388; doi:10.1038/srep26411)
Supplement: Supplementary Dataset 1-3 [file srep26411-s2.doc]

**The Fd-GOGAT1 mutant gene *lc7* confers** **resistance to *Xanthomonas oryzae* pv. *Oryzae* in** **rice**

Honglin Chen^1,2,†^, Chunrong Li^1,†^, Liping Liu^3^, Jiying Zhao^1^, Xuzhen Cheng^2^, Guanghuai Jiang^1^,*, Wenxue Zhai^1^,*

^1^ Institute of Genetics and Developmental Biology, Chinese Academy of Sciences, Beijing 100101, China

^2^ National Key Facility for Crop Gene Resources and Genetic Improvement, Institute of Crop Science, Chinese Academy of Agricultural Sciences, Beijing, 100081, China

^3^ State Key Laboratory of Agrobiotechnology, College of Biological Sciences, China Agricultural University, Beijing, 100193, China

^†^These authors contributed equally to this work.

^*^Corresponding author: Guanghuai Jiang or Wenxue Zhai

Tel: +861064807633; Fax: +861064807633

E-mail: ghjiang@genetics.ac.cn or [wxzhai@genetics.ac.cn](mailto:wxzhai@genetics.ac.cn)

**Supplementary Information**

Supplemental Table S1. The candidate genes in the refine region.

| **Locus identifier** | **Putative function** |
| --- | --- |
| LOC_Os07g46370 | WD domain, G-beta repeat domain contain protein |
| LOC_Os07g46380 | Glycosyltransferase |
| LOC_Os07g46390 | TRAF-type zinc finger domain-containing protein 1 |
| LOC_Os07g46400 | Expressed protein |
| LOC_Os07g46410 | Bifunctional thioredoxin reductase/thioredoxin |
| LOC_Os07g46420 | Expressed protein |
| LOC_Os07g46440 | Ribosomal protein |
| LOC_Os07g46450 | Pleckstrin homology domain-containing protein |
| LOC_Os07g46460 | Erredoxin-dependent glutamate synthase, chloroplast precursor |

Supplemental Table S2. Primers that were used for qRT-PCR.

| Primers | Forward (5'-3') | Reverse (5'-3') |
| --- | --- | --- |
| LOC_Os05g04120 | GGTAGGAGCATCCATCTT | AGCGTGTGAGGTTGTATT |
| LOC_Os08g35210 | TACGATGTTCTTCTCCTTGT | TACGCTCTCCTCTTGTTG |
| LOC_Os09g26660 | AACAGCAAGTAGCAACAAG | CAGCAACCTCATTCATCAC |
| LOC_Os07g46460 | GCATACAAGAACCATCCTAC | CTACTGTCCTTCCATCACT |
| LOC_Os01g27340 | TCGTGTATGAATGCCTCTT | TCGCCATGAAGTAGAAGG |
| LOC_Os03g20840 | TGGAGAGGAAGAAGAATGG | GATGATATGGCAAGTGATGAT |
| LOC_Os01g08370 | GCTCATATCTCCGCTCAT | TCTCAACAGGTCACATCC |
| LOC_Os01g65920 | GGCTGGTGTGATAGTGTA | CCACGCTCTCATCTGTTA |
| LOC_Os12g13550 | GGTGATTGACTCTGTTCTTG | GCTTGTGATGTTGTTACTGA |
| LOC_Os03g21710 | GAATTACGACATCACCTACTAC | CGAGACGAATTAGCAACAA |
| LOC_Os04g51560 | CACTGCTCCAAGAGAAGG | ACTTCCTCCACGAGTATTC |
| LOC_Os07g48260 | ATGAAGTTATGGACGACCT | GATCCTCTTCTTCCTCCTC |
| LOC_Os12g02470 | ATCAATGGAGGAAGTATGGT | AGTATGCTCTGAGATGTAGG |
| LOC_Os05g49620 | GGCTACTTCTCCTTCATCT | TTCTGAATCTCCGATTGGA |
| LOC_Os05g39720 | TACTCGGACTTCACGTTC | ATGGTCGGTTCTTGGTAG |
| LOC_Os03g32230 | GAACTTCGACCTCAACCT | GGACATCCTGAGCTTCTT |
| LOC_Os05g02390 | CTCGACCTGAACCATCCA | CTCCGGTATCCAAGAACTG |
| LOC_Os02g53130 | AAGAAGAGCCTGTCCTAC | CCTCGCCCTTGAAGTATA |
| LOC_Os02g02210 | CAAGCAGTGGTTAAGAAGTAT | CAAGCAGTGGTTAAGAAGTAT |
| LOC_Os03g18130 | CGACAAGGAGTTCATCAAC | GCAGCGTTCTTCATCATT |
| LOC_Os08g02030 | GAACACATGGTCGGAGAT | GAGAAAGCGAAGAAGCATT |
| LOC_Os04g38680 | GATCTACACGACGCTGAT | TTGACGGACCTCTTGTTG |
| LOC_Os03g40540 | GTTGAAATGAATGGGTATGTTATC | TTCTACGGTGCCTACTTC |
| LOC_Os11g18570 | CGTAGGAGCACACTCATAT | CTATTGATGGACTTGTTCTGAA |
| LOC_Os11g25160 | GGAGCAATGAACCAAGTC | TCAGATAGGAGCGGAGTA |
| LOC_Os01g01660 | AGAACAAGACGGTGAACA | ACTCCTGGATTTGCTTGA |
| LOC_Os07g02440 | ATGATGGTGGTGGTGATG | GAAGCAGTCGTGGAAGAA |
| LOC_Os04g41260 | TAATGTCCAGAGGATGAAGTT | AGAGCAATGTAGCAAGGT |
| LOC_Os05g10930 | GAGAGGATCGGGAGGCTG | GAGGTGGAGGCCCATGAG |
| LOC_Os12g43380 | GGACTTCTACGACCTGTC | TGTGTCTTGGTGTTGTCT |
| LOC_Os10g35070 | CTCGCCGATTATGTTCAC | GCAGTTATCATACTTGAGGTAG |
| LOC_Os01g71670 | GGAGTTGCTTCTATGCTTAC | GATGCCCTTGGACTTGTA |
| LOC_Os01g24710 | GGACAGGAATATGCCATTG | CCAGCCTCGTATGTATTATTAG |
| LOC_Os02g35329 | TGATCGAGATCCCCGAAT | CAGAGCCTTCTCAGTGAC |
| LOC_Os04g21130 | GAGGTCTCGTCTGGATAC | TTGTGTTGAAGATACCATAGATG |
| LOC_Os07g43240 | AAGAAGGTGGTGTTGGAT | CGAAGTGCTTGTTCATGT |
| LOC_Os08g31140 | GCACTACCTGTCTGATCT | CAGTCCATCCTCACCTTC |
| LOC_Os10g29650 | AAGGCTATCGCTGTTATGA | AGAAGGCTGATTAGTAGAAGAA |
| LOC_Os04g45920 | CCATCAGAAGGTCAGACA | GGATCTCGTACAGCACAA |
| LOC_Os01g06310 | CTGGTCTCCAAGCTCGTC | CCGTGCTGTGGAGGATAC |
| LOC_Os01g01660 | AGAACAAGACGGTGAACA | ACTCCTGGATTTGCTTGA |
| LOC_Os01g01880 | AAGCAATTAAGCAGGAAGG | ATCAATCGTCTCCGTCTC |
| LOC_Os01g06310 | CTGGTCTCCAAGCTCGTC | CCGTGCTGTGGAGGATAC |
| LOC_Os01g34970 | CTGAAGTATGAAGAAGCAAGT | AAGTAGAACACAAGGAAGGA |
| LOC_Os01g48360 | CATGAACCAGAGGCTCAG | CTAGGTGATCATCAGTCGG |
| LOC_Os01g71670 | GGAGTTGCTTCTATGCTTAC | GATGCCCTTGGACTTGTA |
| LOC_Os02g42330 | GACTTGGACCTTGGAGAA | TCCTAGAGATACCGTCACT |
| LOC_Os03g18130 | CGACAAGGAGTTCATCAAC | GCAGCGTTCTTCATCATT |
| LOC_Os04g21230 | ATGGATGTCCTGGAGTTC | GATGTAGCGGTTCAACAC |
| LOC_Os04g45920 | CCATCAGAAGGTCAGACA | GGATCTCGTACAGCACAA |
| LOC_Os07g01340 | GTGGAGCAACGATGTGTA | GATGATGAGCGTGTGGTA |
| LOC_Os07g02440 | ATGATGGTGGTGGTGATG | GAAGCAGTCGTGGAAGAA |
| LOC_Os07g03730 | GAGAAGCAGTGGTACGAC | AGTAGTTGCAGGTGATGAA |
| LOC_Os08g31140 | GCACTACCTGTCTGATCT | CAGTCCATCCTCACCTTC |
| LOC_Os10g29650 | AAGGCTATCGCTGTTATGA | AGAAGGCTGATTAGTAGAAGAA |
| LOC_Os10g35070 | CTCGCCGATTATGTTCAC | GCAGTTATCATACTTGAGGTAG |
| LOC_Os11g25160 | GGAGCAATGAACCAAGTC | TCAGATAGGAGCGGAGTA |
| LOC_Os11g37970 | AAGTGTATCCAGGTGAAGAA | TGAAGATGGTCTCGTAGTC |
| LOC_Os12g43380 | GGACTTCTACGACCTGTC | TGTGTCTTGGTGTTGTCT |
| LOC_Os01g01880 | AAGCAATTAAGCAGGAAGG | ATCAATCGTCTCCGTCTC |
| LOC_Os01g48360 | CATGAACCAGAGGCTCAG | CTAGGTGATCATCAGTCGG |

Supplemental Table S3. Primers that were used for vector construction.

| Primers | Forward (5'-3') | Reverse (5'-3') |
| --- | --- | --- |
| *FdGOGAT-*C | CTCGAGATGGCCACGCTCCCACGTGC | GAGCTCTCACTTCGCCGATTGTACTG |
| *FdGOGAT-*O | GAGCTCATGGCCACGCTCCCACGTGC | GTCGACCTTCGCCGATTGTACTGTTG |
